# Clinical Assessment of Ultrasound-Guided Local Lauromacrogol Injection Combined With Curettage and Hysteroscopy for Cesarean Scar Pregnancy

**DOI:** 10.3389/fphar.2020.601977

**Published:** 2020-12-21

**Authors:** Qing Wu, Xia Liu, Lin Zhu, Yichen Zhu, Tingting Mei, Shanshan Cao, Yan Shen, Jun Ding, Tan Lin

**Affiliations:** ^1^ Department of Gynecology, Zhejiang Provincial Peoples’ Hospital, People’s Hospital of Hangzhou Medical College, Hangzhou, China; ^2^ Department of Obstetrics and Gynecology, Fujian Provincial Hospital, Clinical Medical School of Fujian Medical University, Fuzhou, China; ^3^ Zhejiang University School of Medicine, Hangzhou, China; ^4^ Tiantai People’s Hospital of Zhejiang Province, Zhejiang, China

**Keywords:** curettage, hysteroscopy, uterine artery embolization, cesarean scar pregnancy, lauromacrogol

## Abstract

**Background:** To evaluate the efficacy, safeness and cost of ultrasound-guided local lauromacrogol injection (USG-LLI) combined with curettage and hysteroscopy for cesarean scar pregnancy (CSP).

**Methods:** This was a retrospective study included 151 CSP patients diagnosed with CSP from June 2017 to December 2019, and treated by USG-LLI (*n* = 86) or uterine artery embolization (UAE) (*n* = 65) combined with curettage and hysteroscopy. Clinical data and outcome were analyzed.

**Results:** There were no significant differences in basic clinical characteristics in the two groups. Two groups showed the similar success rates. USG-LLI group, compared with UAE group had significantly lower complication rates (9.30 vs. 44.62%), lower total costs (both medical and non-medication cost) (*p* < 0.05).

**Conclusions:** USG-LLI combined with curettage and hysteroscopy is a feasible method to treat CSP with minimal invasion and high efficacy. Moreover, compared with curettage after UAE, USG-LLI exhibited lower complication rate and required fewer expenses.

## Introduction

Cesarean scar pregnancy (CSP) is a rare type of ectopic pregnancy characterized by an empty uterus and cervical canal, and a gestational sac (GS) located in the anterior uterine scar of cesarean section with thin myometrium between the sac and the bladder (Fylstra, 2002).

It is recently estimated that 1 in 531 women with a cesarean scar will have a CSP and that 4.2% of ectopic pregnancies are CSP (Singh et al., 2012). CSP may lead to life-threatening complications, such as massive vaginal bleeding and uterine rupture, because of the thin muscle wall the scar has, which reduces the contractility of the uterus.

At present, there is no consensus on the standardized therapy for CSP in clinical practice (Petersen et al., 2016). Common treatment methods include drug therapy, curettage under the ultrasound, uterine artery embolization (UAE), and operative hysteroscopy followed by curettage. It should be noted that an effective treatment not only should prevent the occurrence of severe blood loss, but also preserve fertility function, women’s health, and quality of life (Gao et al., 2018; Kim and Moon, 2018).

Blocking the blood flow to the sac through the method of uterine artery embolization (UAE) results in the death of the embryo and reduces bleeding during curettage. UAE followed by curettage has been applied in the treatment of CSP for its minimal invasion and efficiency, but it may incur uncomfortable sensation of fever, leg pain, and postembolization syndrome (Tumenjargal et al., 2018).

Lauromacrogol (Polidocanol; Polyoxyethylene 10 laurylether) is a foamed sclerosant drug and is the most commonly used sclerosant (Dong et al., 2019). It has been widely applied in sclerotherapy of uterine myomas, hemangioma, varicose vein of the lower extremities, and cyst disease (Eckmann, 2009; Zhou et al., 2014).

We supposed that local lauromacrogol injection could block the veins around gestational sac located at the cesarean scar, kill the embryo and prevent hemorrhage during curettage. Here we introduce the treatment for CSP and investigate the efficacy and cost of ultrasound-guided local lauromacrogol injection (USG-LLI) combined with curettage and hysteroscopy for CSP compared to UAE pretreatment.

## Materials and Methods

### Study Patients

This retrospective study was approved by the ethics committee of Zhejiang Provincial Peoples’ Hospital (2019KY291). From June 2017 to December 2019, this study collected 151 CSP patients from Department of Gynecology in Fujian provincial hospital, Department of Gynecology in Zhejiang Provincial Peoples’ Hospital, the two hospitals were the central hospitals which accepted many patients from primary hospitals in each province.

Patients with a ruptured uterus, massive vaginal bleeding, abnormal coagulation, lack of myometrial layer between the bladder and sac, severe cardiac, lung, kidney, or liver disease, acute inflammation, or an allergy to lauromacrogol were excluded. Patients with a sac larger than 60 mm were excluded from this study.

The two centers had both techniques, and all patients accepted informed consent and were informed of the benefits and potential risks of each methods, and patients selected the method by themselves. The medical records and ultrasound of all patients with CSP were collected from the original medical records and clinical interview follow-ups.

### Diagnosis

The diagnosis of CSP was made by the history of cesarean delivery and serum β-hCG (β-human chorionic gonadotropin) and we confirmed patients with CSP using the following transvaginal ultrasonographic criteria (Timor-Tritsch et al., 2012): 1) an empty uterine cavity and cervical canal, without contact with the sac; 2) a gestational sac at the anterior wall of the isthmic segment with or without cardiac activity; 3) a visible myometrial defect between the bladder and the sac; 4) functional trophoblastic/placental circulation surrounding the gestation sac/mass.

### UAE Combined With Curettage and Hysteroscopy

UAE with local MTX infusion before curettage was performed. We selected the right transfemoral approach for artery access, and the uterine artery was selectively catheterized with a 5F Yashiro catheter (TERUMO, Tokyo, Japan) and embolized with 1,000–1,400 μm sized gelatin sponge particles (Alicon Co. Ltd., Hangzhou, China), with 25 mg methotrexate (MTX) infused into each uterine artery bilaterally prior to the embolization procedure. After embolization, angiography was conducted to confirm whether the occlusion of the blood flow was finished. Traditional curettage were performed first and then hysteroscope checked 24–72 h later by qualified doctors.

### Ultrasound-Guided Local Lauromacrogol Injection (USG-LLI) Combined With Curettage and Hysteroscopy

A 21-gauge needle was used (Hakko, Tokyo, Japan) to inject the lauromacrogol, and the location of the gestational sac was identified for injection under transvaginal ultrasound. 5–10 ml lauromacrogol (Lauromacrogol Injection, 10 ml: 100 mg; Tianyu Pharmaceutical Co. Ltd., Shanxi, China) was slowly injected at several points around the peritrophoblastic tissue until rare blood flow was detected peripherally. 12–24 h later, after the vascularity of the lesion completely obstructed, curettage and hysteroscopy were performed by qualified doctors. Before lauromacrogol injection and hysteroscopy, transvaginal contrast-enhanced ultrasonography by sulfur hexafluoride microbubble (Bracco, Milan, Italy) was performed individually to examine the blood supply. Specimens were sent for pathological examination.

### Cost Analysis

Both direct and indirect costs were calculated in our study. The direct costs included the cost of medication, patient care, clinical procedure, nursing, ward, clinical material and the non-medical costs. The non-medical costs included cost of transportation and meals. The indirect costs included loss of working time of the patients and accompanying persons. Both hospitals were used the same standardized cost for patients.

### Treatment Assessment and Follow-Up

A successful treatment was defined as complete recovery without severe complications (such as massive bleeding, gastrointestinal perforation and uterine rupture), without second line therapy (Chai et al., 2018).

The failure of treatment was defined as complications such as vaginal bleeding of more than 200 ml, a decrease in the serum β-hCG level by ≤50% for the seventh day, or treated by second line therapy such as Foley balloon catheter insertion, laparoscopy, or laparotomy (Xiao et al., 2017).

All patients were observed, and estimated blood loss and side effects (including fever, vomiting, vaginal bleeding and pain) were recorded. All patients were requested to abstain from intercourse until CSP resolution. The dynamic levels of serum β-hCG were determined weekly until the levels decreased to the normal level (cutoff was 5 IU/L). Ultrasound was used to monitor the resolution of the retained mass after normalization of serum β-hCG level. Patients’ menstruation recovery was recorded. After complete recovery, the thickness of endometrium and uterine adhesion of all patients were assessed by ultrasound during luteal phase after 3–6 months.

### Statistical Analysis

Mean ± standard deviation was presented for continuous and ordinal data; categorical data were presented as the count and percentage. Before the two-sample *t* test, the examined features had been confirmed the normal distribution. All demographic and baseline clinical variables were examined with two-sample t tests. Independent sample t tests were used to examine differences between UAE group and USG-ILL group on outcomes after treatments as well as costs. The Chi-square test was used to compare categorical data. For categorical data, fisher-exact test was applied if *n* < 5.

Non-parametric test was used for continuous variable. SPSS 17.0 software (SPSS Inc., Chicago, IL, United States) was used for statistical analysis. Additionally, all statistical tests were two tailed and a *p* value <0.05 was considered statistically significant.

## Results

### Demographic and Baseline Clinical Characteristics of Women With CSP

A total of 151 CSP patients were enrolled in this retrospective study. 65 women underwent UAE combined with uterine curettage and hysteroscopy, and 86 patients underwent USG-LLI combined with uterine curettage and hysteroscopy. All patients had previous cesarean delivery. The demographic data and baseline clinical characteristics of participants are summarized in Table 1.

**TABLE 1:** Demographic and baseline clinical characteristics of participants prior to curettage.

Characteristic	UAE group (*n* = 65)	USG-LLI group (*n* = 86)	*p*
Patient age (years)	34.00 ± 3.81	33.17 ± 3.53	0.171
No. of previous cesarean sections	1.63 ± 0.49	1.57 ± 0.60	0.506
Interval time from recent cesarean section (years)	4.96 ± 3.30	5.82 ± 2.97	0.095
Gestational age (d)	50.48 ± 9.12	48.51 ± 12.84	0.296
Fetal heart activity(n)	51	65	0.678
Serum β-hCG (U/L)	20,977.23 ± 16,231.89	26,981.45 ± 24,444.67	0.089
Diameter of the sac (mm)	29.17 ± 8.61	26.19 ± 11.38	0.08
Thickness of myometrium (mm)	2.02 ± 0.73	2.14 ± 0.9	0.383

There were no statistically significant differences on age, gestational age, interval time from recent caesarean section, fetal heart activity rate, serum β-hCG, diameter of the sac and the thickness of myometrium before the treatment in the two groups (Table 2).

**TABLE 2:** Outcome of women with CSP treated with UAE or with USG-LLI prior to curettage.

Variables	UAE group (*n* = 65)	USG-LLI group (*n* = 86)	*p*
Success rate (%)	64/65	85/86	0.842
Complication rate (%)	29/65	8/86	0.000
Abdominal/pelvic pain	14	4	0.002
Leg pain	1	0	0.248
Fever	14	4	0.002
Time to β-hCG Normalization(d)	32.48 ± 10.08	30.49 ± 7.17	0.159
Duration of hospital Stay(d)	4.58 ± 1.78	4.64 ± 2.02	0.862
Blood Loss (ml)	28.62 ± 64.14	22.32 ± 25.48	0.409
0–199 ml	64	85	0.842
≥200 ml	1	1	0.842
Blood transfusion	1	0	0.248
TE (mm)	8.48 ± 1.93	7.89 ± 2.19	0.084
Restored menses(n)	64	86	0.248
Intrauterine adhesions(n)	1	0	0.248

TE, Thickness of endometrium during luteal phase after treatment.

### Sclerotherapy Blocked the Blood Flow Around the Gestational Sac

**FIGURE 1:**
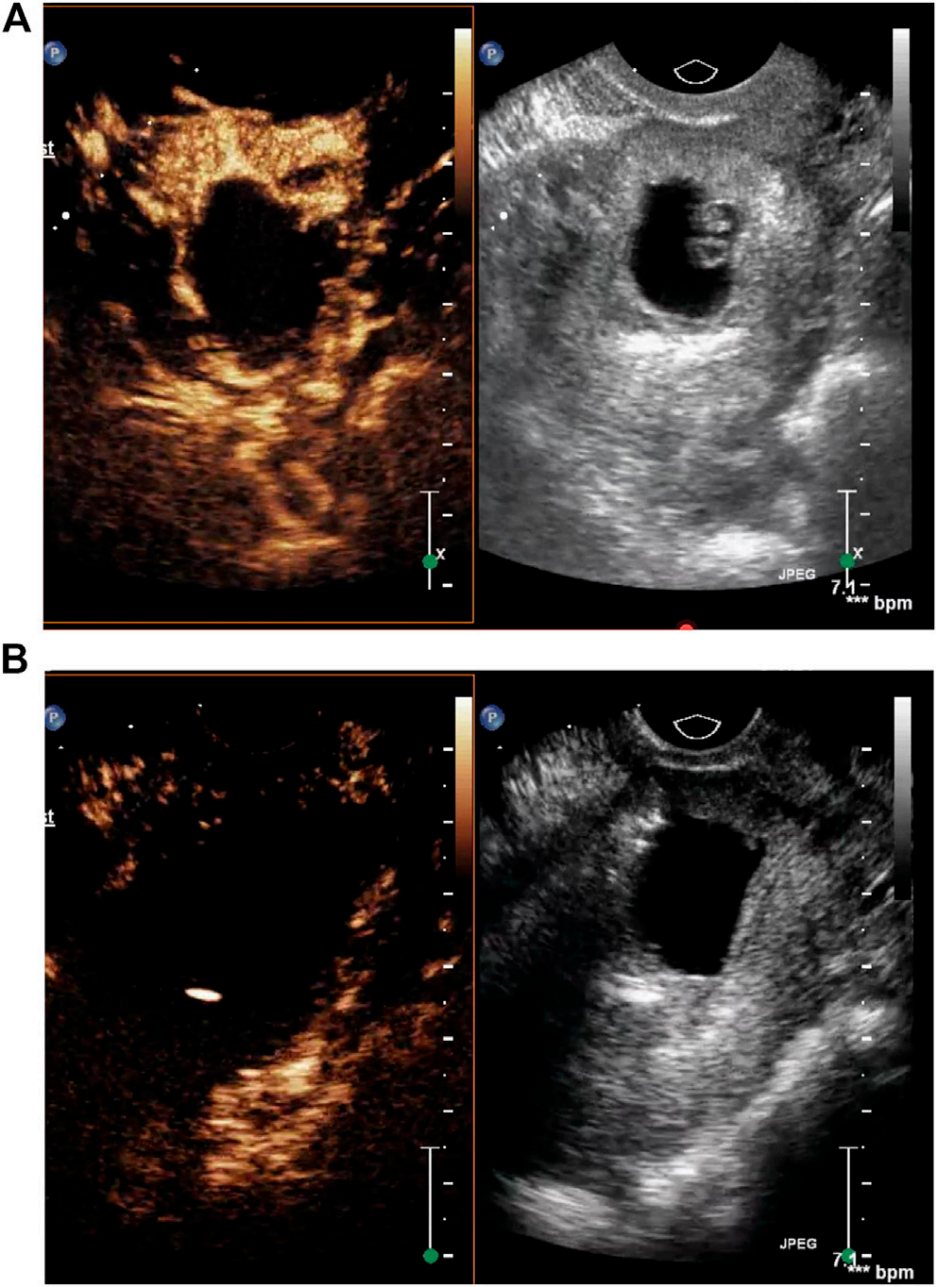
**(A)** The contrast-enhanced ultrasound picture indicated abundant blood supply in the area surrounding the gestational sac. **(B)** Transvaginal contrast-enhanced ultrasonography before curettage showed that the blood flow in the peripheral tissue of the pregnancy sac was completely occluded.

Before lauromacrogol injection, we injected 5 ml sulfur hexafluoride microbubble, which serves as contrast agent, intravenously into the patient. The contrast-enhanced ultrasound picture (Figure 1A) indicated abundant blood supply in the area surrounding the gestational sac. Before curettage and hysteroscopy, transvaginal contrast-enhanced ultrasonography was performed again to examine the blood supply. And it turned out that the blood flow in the peripheral tissue of the pregnancy sac was completely occluded (Figure 1B). Therefore, lauromacrogol injection was sufficient to cause aseptic inflammatory lesion and fibrosis, lowering the risk of complications such as massive bleeding during curettage.

### Outcome of Women With CSP Treated With UAE or With USG-LLI Prior to Curettage

No patients were lost to follow-up. There were no significant differences in time to β-hCG normalization, duration of hospital stay, blood loss and blood transfusion rate during the treatment and follow-up in the two groups (Table 2). The maximal blood loss of UAE group was 500 ml, while the maximal blood loss of USG-LLI group was 200 ml.

The success rates of the two groups were similar, while the complication rates (24.62%, 5.81%) of two groups were different significantly (Table 2). In the UAE group, after embolization, 29 patients presented complications, of which 14 patients had a low-grade fever lasting 1–3 d, 1 patients had leg pain, and 14 patient had pelvic pain. Both of them needed pain killer to alleviate it. In the USG-LLI group, only four patients had a low-grade fever, which was self-limiting without medication (Table 2). After the operation, we tested the thickness of endometrium and intrauterine adhesions by ultrasound scanning during luteal phase 3 months later.

### Cost Analysis of Women With CSP Treated With UAE or With USG-LLI Prior to Curettage

As is illustrated in Table 3, in both groups, the non-medication cost accounted for the largest part of the total cost. As we compared total cost between two groups, we found that the cost was significantly higher in UAE group (*p* < 0.05). We also found that the non-medication cost and direct medical cost of UAE group were higher than those in USG-LLI group (*p* < 0.05) (Table 3).

**TABLE 3:** Cost analysis of women with CSP treated with UAE or with USG-LLI prior to curettage.

Variable	UAE group (*n* = 65)	USG-LLI group (*n* = 86)	*p*
Direct medical cost (yuan)	16,485.23 ± 3,980.41	7,518.03 ± 1,365.57	0.000
Medication cost (yuan)	2073.51 ± 791.83	2,147.62 ± 544.11	0.497
Non-medication cost (yuan)	14,133.23 ± 3,981.63	5,099.25 ± 955.55	0.000
Non-medical cost (yuan)	278.65 ± 67.78	271.10 ± 45.48	0.415
Indirect cost (yuan)	304.01 ± 60.79	318.03 ± 50.82	0.126
Total cost (yuan)	16,789.32 ± 3,976.33	7,836.01 ± 1,371.81	0.000

## Discussion

With the rate of cesarean delivery increasing particularly in China, the rate of CSP keeps ascending (Lan et al., 2013). As the villus of CSP adheres to the mesometrium, it gradually implants into it, which may eventually lead to penetration. Life-threatening complications could happen if there is not a timely appropriate treatment.

The villus implanted with tissue in a cesarean scar is difficult to remove because of the connective tissue within the thin mesometrium layer. Meanwhile, for the lack of muscle fiber, it is difficult for the scar to contract effectively. Thus, curettage without prophylactic treatment could cause massive blood loss (Khunda and Tay, 2007). Currently, there is no uniform treatment for CSP (Petersen et al., 2016). Excision of the gestational sac and repairing the uterine defect using laparoscopy or laparotomy seems to reduce complications and risk of future recurrence. However, it results in long hospitalization time, high cost, long-term recovery, and quite some adverse reaction.

UAE has been widely used in the pretreatment of CSP in the past decade (Cao et al., 2014; Qian et al., 2015). It blocks the blood flow in the uterine arteries for efficient reduction of bleeding risk during curettage. UAE combined with curettage may become a safe and effective treatment for CSP. Nonetheless, it is UAE that can cause serious complications, such as puncture point hematoma, lower limb arterial embolism, vascular rupture. It can even lead to pulmonary embolism, which is rare yet life-threatening. Meanwhile, uterine ischemia after UAE can cause spastic pain, nausea, vomiting, fever, and even ovarian insufficiency (Arthur et al., 2014; Petersen et al., 2016). In this study, we found that there were more patients suffered from fever and pain in the UAE group compared to USG-LLI group. Although the UAE group had high efficiency to block the vessels and reduce the blood loss during curettage and hysteroscopy, it required sophisticated and advanced digital imaging apparatus, which was expensive for Chinese primary hospitals and patients to afford.

Lauromacrogol is another type of clinical chemical sclerosants different from alcohol. It has been widely used in sclerotherapy. The target of Lauromacrogol as a sclerosant is endothelial cells (ECs). Lauromacrogol exerts its sclerosant functions by two pathways. First, paravenous lauromacrogol injection can induce fibrosis in the veins around the targeted site causing vascular compression and hemostasis. Second, direct intravascular lauromacrogol injection can destroy endothelial cells within the targeted vessels, thus promoting local thrombogenesis. The occlusion of vessel lumen can result in an aseptic inflammatory lesion and tissue fibrosis, and the impaired veins may eventually develop into fibrous cords (Eckmann et al., 2005; Parsi et al., 2008). With increasing number of innovative and less-invasive treatment approaches emerging, sclerosing agents have been frequently used in gynecological operations, such as ovarian cyst and uterine fibroids (Fei et al., 2018; Bao et al., 2020).

In this study, we recorded the clinical information including the outcome and the cost of each treatment. We found that USG-LLI combined curettage and hysteroscopy was minimally invasive and highly efficient. Compared with curettage after UAE, USG-LLI resulted in lower complication rate and required less expense. Ultrasound-guided local lauromacrogol injection combined with curettage and hysteroscopy is an effective treatment for patients without emergent situation such as massive vaginal bleeding. The likelihood of incorrect application is very low, since surgeons slowly inject about 5–10 ml Lauromacrogol into multiple points (instead of targeted vessel) around the gestational sac, which reduces the dosage in each point, thus minimizing the risk of damage in adjacent tissues. Meanwhile, this process does not require advanced devices, whose cost is much lower than UAE. when ultrasound indicated rare blood flow peripherally, the peri-trophoblastic tissue was considered sclerosed, and about 12–24 h later, curettage and hysteroscopy can be performed to remove tissue. Hence, it is quite feasible for primary hospital without expensive equipment for vascular intervention to perform USG-LLI surgeries.

But it was difficult to predict future risk of recurrence and fertility affection. Some limitations of our study were as follows. This was a retrospective study and reported limited data on subsequent pregnancy outcomes after cesarean scar pregnancy. It requires large-sample clinical studies and a long term to follow up the patients who have the desire of future pregnancy. Since there is rare study about USG-LLI, it need more exploration to confirm the indication, complication and contraindication about this procedure.

We conclude that USG-LLI or UAE combined with curettage and hysteroscopy were both effective for CSP. While the complications followed UAE may be more than USG-LLI, and the costs of UAE were higher than those of USG-LLI. Therefore, the overall benefit and risk should be evaluated rationally to achieve the maximum benefit for an individual. USG-LLI combined with curettage and hysteroscopy for CSP is safe and effective and the cost is lower.

## Data Availability Statement

The original contributions presented in the study are included in the article/Supplementary Material, further inquiries can be directed to the corresponding author.

## Ethics Statement

Written informed consent was obtained from the individual(s) for the publication of any potentially identifiable images or data included in this article.

## Author Contributions

QW, YZ, and TL conceived the study and participated in its design, drafting and writing the manuscript as well as supervising the study and critically revising the manuscript. TM, SC, LZ, and XL collected the clinical data. QW, YZ, YS, and JD was responsible for drafting and writing the manuscript and statistical analysis. All authors substantially contributed to the revision of the manuscript.

## Conflict of Interest

The authors declare that the research was conducted in the absence of any commercial or financial relationships that could be construed as a potential conflict of interest.

## References

[B1] Arthur R. Kachura J. Liu G. Chan C. Shapiro H. (2014). Laparoscopic myomectomy versus uterine artery embolization: long-term impact on markers of ovarian reserve. J. Obstet. Gynaecol. Can 36, 240–247. 10.1016/s1701-2163(15)30632-0 24612893

[B2] Bao W. Liao H. Zhang Q. (2020). Clinical efficacy of ultrasound-guided radiofrequency ablation and lauromacrogol sclerotherapy for uterine fibroids. Int. J. Clin. Exp. Med 13, 1678–1686.

[B3] Cao S. Zhu L. Jin L. Gao J. Chen C. (2014). Uterine artery embolization in cesarean scar pregnancy: safe and effective intervention. Chin. Med. J 127, 2322–2326. 10.3760/cma.j.issn.0366-6999.20140196 24931250

[B4] Chai Z. Y. Yu L. Liu M. M. Zhu T. W. Qi F. (2018). Evaluation of the efficacy of ultrasound-guided local lauromacrogol injection combined with aspiration for cesarean scar pregnancy: a novel treatment. Gynecol. Obstet. Invest 83, 306–312. 10.1159/000485099 29208846

[B5] Dong Y. Zhou J. Liu Z. Luo T. Zhan W. (2019). Efficacy assessment of ultrasound guided lauromacrogol injection for ablation of benign cystic and predominantly cystic thyroid nodules. Front. Pharmacol 10, 478 10.3389/fphar.2019.00478 31139077 PMC6517687

[B6] Eckmann D. M. Kobayashi S. Li M. (2005). Microvascular embolization following polidocanol microfoam sclerosant administration. Dermatol. Surg 31, 636–643. 10.1111/j.1524-4725.2005.31605 15996412

[B7] Eckmann D. M. (2009). Polidocanol for endovenous microfoam sclerosant therapy. Expet Opin. Invest. Drugs 18, 1919–1927. 10.1517/13543780903376163 PMC2787977 19912070

[B8] Fei D. Li Y. Sui G. (2018). “Clinical study of ultrasound-guided puncture lauromacrogol sclerosis in the treatment of ovarian endometriosis cyst,” in 3rd international Conference on Materials Science, Resource and Environmental Engineering, Changsha City, China, October2018 Editors You Z. Xiao J. Tan Z.

[B9] Fylstra D. L. Pound-Chang T. Miller M. G. Cooper A. Miller K. M. (2002). Ectopic pregnancy within a cesarean delivery scar: a case report. Am. J. Obstet. Gynecol 187, 302–304. 10.1097/00006254-200208000-00024 12193916

[B10] Gao L. Hou Y. Y. Sun F. Xia W. Yang Y. Tian T. (2018). A retrospective comparative study evaluating the efficacy of adding intra-arterial methotrexate infusion to uterine artery embolisation followed by curettage for cesarean scar pregnancy Arch. Gynecol. Obstet 297, 1205–1211. 10.1007/s00404-018-4686-8 29497822

[B11] Khunda A. Tay J. (2007). Caesarean scar pregnancy. BJOG 114, 1304 10.1111/j.1471-0528.2007.01436.x 17877690

[B12] Kim Y. R. Moon M. J. (2018). Ultrasound-guided local injection of methotrexate and systemic intramuscular methotrexate in the treatment of cesarean scar pregnancy. Obstet. Gynecol. Sci 61, 147–153. 10.5468/ogs.2018.61.1.147 29372162 PMC5780311

[B13] Lan W. Hu D. Li Z. Wang L. Yang W. Hu S. (2013). Bilateral uterine artery chemoembolization combined with dilation and curettage for treatment of cesarean scar pregnancy: a method for preserving the uterus. J. Obstet. Gynaecol. Res 39, 1153–1158. 10.1111/jog.12051 23718134

[B14] Parsi K. Exner T. Connor D. E. Herbert A. Ma D. D. Joseph J. E. (2008). The lytic effects of detergent sclerosants on erythrocytes, platelets, endothelial cells and microparticles are attenuated by albumin and other plasma components *in vitro* . Eur. J. Vasc. Endovasc. Surg 36, 216–223. 10.1016/j.ejvs.2008.03.001 18396426

[B15] Petersen K. B. Hoffmann E. Larsen C. R. Nielsen H. S. (2016). Cesarean scar pregnancy: a systematic review of treatment studies. Fertil. Steril 105, 958–967. 10.1016/j.fertnstert.2015.12.130 26794422

[B16] Qian Z. D. Huang L. L. Zhu X. M. (2015). Curettage or operative hysteroscopy in the treatment of cesarean scar pregnancy. Arch. Gynecol. Obstet 292, 1055–1061. 10.1007/s00404-015-3730-1 25935196

[B17] Singh K. Soni A. Rana S. (2012). Ruptured ectopic pregnancy in caesarean section scar: a case report. Case. Rep. Obstet. Gynecol, 2012, 106892 10.1155/2012/106892 23198194 PMC3502781

[B18] Timor-Tritsch I. E. Monteagudo A. Santos R. Tsymbal T. Pineda G. Arslan A. A. (2012). The diagnosis, treatment, and follow-up of cesarean scar pregnancy. Am. J. Obstet. Gynecol 207, 44–13. 10.1016/j.ajog.2012.04.01844 22607667

[B19] Tumenjargal A. Tokue H. Kishi H. Hirasawa H. Taketomi-Takahashi A. Tsushima Y. (2018). Uterine artery embolization combined with dilation and curettage for the treatment of cesarean scar pregnancy: efficacy and future fertility. Cardiovasc. Interv. Radiol 41, 1165–1173. 10.1007/s00270-018-1934-z 29546456

[B20] Xiao J. Shi Z. Zhou J. Ye J. Zhu J. Zhou X. (2017). Cesarean scar pregnancy: comparing the efficacy and tolerability of treatment with high-intensity focused ultrasound and uterine artery embolization. Ultrasound Med. Biol 43, 640–647. 10.1016/j.ultrasmedbio.2016.11.001 27979666

[B21] Zhou X. Xie Y. Cui Q. Xu X. He M. Liang B. (2014). [Safety and efficacies of ultrasound-guided sclerotherapy of uterine fibroids with lauromacrogol]. Zhonghua Yi Xue Za Zhi 94, 2204–2206. 10.3760/cma.j.issn.0376-2491.2014.28.011 25331473

